# Prompting consumers to make healthier food choices in hospitals: a cluster randomised controlled trial

**DOI:** 10.1186/s12966-020-00990-z

**Published:** 2020-07-06

**Authors:** Julia L. Allan, Daniel J. Powell

**Affiliations:** 1 Health Psychology, Institute of Applied Health Sciences, Health Sciences Building, University of Aberdeen, Foresterhill, Aberdeen, AB25 2ZD UK Scotland; 2 Rowett Institute, University of Aberdeen, Foresterhill, Aberdeen, AB25 2ZD UK Scotland

**Keywords:** Hospital, Food choice, Snack, Healthy, Point of purchase prompt, Nudge

## Abstract

**Background:**

Hospitals in the UK offer snacks for sale to patients, staff and visitors. Despite the NHS’s health promoting role, and tightening of regulations around which foods can be sold in hospitals, many snacks purchased in this setting are unhealthy. The present project tests the effectiveness of theory-based point of purchase prompts (PPPs; a form of cognitive nudge) designed to make it cognitively easier for consumers to compare available products and choose healthier options.

**Methods:**

Hospital shops in Scotland (*n* = 30) were recruited into a cluster randomised controlled trial to test whether a PPP could reduce the average calorie, fat and/or sugar content of purchased snacks. Inclusion criteria stated that eligible sites; sold food; were located in a hospital; and were accessible to staff, patients and visitors. The PPP intervention was a theory-based sign (tailored to the available range in each location) designed to cognitively simplify healthier snack choices by facilitating cross-product comparison. Shops were randomised to display PPPs (intervention; *n* = 15) or not (control; *n* = 15) using block randomisation controlling for shop size. Data on all snacks purchased from participating shops were obtained from retailers for a 12-week baseline and 12-week follow-up period. Primary outcomes were the average calorie (kcals), fat(g) and sugar(g) content of snacks purchased each day. Secondary outcomes were the average customer spend per item purchased (£,p) and the total number of snacks purchased daily. Shop staff were not blinded to group assignment but data providers were. Data were analysed using mixed effects multi-level regression models.

**Results:**

Data from > 1 million snack purchases were analysed. Snacks purchased from intervention sites were on average significantly lower in calorie (γ = − 1.84, *p* < .001) and sugar (γ = − 0.18, *p* = .030) at follow up relative to baseline but only the reduction in calories was significantly different to control. Average spend per item also reduced significantly in intervention (but not control) sites (γ = − 0.89, *p* < .001). The intervention had no effect on the fat content of snacks or the number of snacks sold.

**Conclusions:**

Simple, theory-based point of purchase prompts can produce small but statistically significant reductions in the energy content of snack purchases from hospital shops.

**Trial registration:**

Retrospectively registered (8/Oct/2018) with ISRCTN (ID: ISRCTN90365793).

## Background

Unhealthy dietary choices and patterns are consistently related to weight gain and poor health outcomes [1–3] and are a key target for public health intervention. Many unhealthy food choices are made outside of the home [4] with little or no conscious awareness [5–7] and are strongly related to features of the environment such as food availability [8].

People have strong innate preferences for foods that are high in fat and/or sugar [9] and limited cognitive and motivational resources with which to resist temptation [8]. Consequently, when unhealthy foods are readily available in the surrounding environment, they are likely to be opportunistically selected and consumed [10]. Availability is one of six core features of the proximal environment identified as having the potential to elicit health behaviour change when modified [11]. Numerous empirical studies have demonstrated that consumers are more likely to choose healthy options when such options are (relative to unhealthy options) more readily available within an environment. For example, Van Kleef and colleagues [12] demonstrated in both a controlled laboratory experiment and an observational field study of snack choices in a hospital canteen that consumers chose healthier options when the available range was modified to contain 75% rather than 25% healthy options. Similarly, a recent systematic review of healthy choice interventions in vending machines [13] demonstrated that interventions which altered availability were effective in increasing selection of healthy options.

On the back of such evidence, researchers, policy makers and public health professionals have become increasingly interested in structural changes which can be made to public food environments in order to support population level changes in food choice and consumption. Much of this interest has focused on public settings with an underlying responsibility for health such as hospitals.

In addition to clinical services, most hospitals contain a selection of food retail units (from vending machines to shops, cafes and canteens) which sell food and drinks to patients, staff and visitors. Food retail units in hospitals have four features which make them attractive settings in which to host food choice interventions. Firstly, hospitals offer a relatively unique opportunity to deliver health interventions to a large and diverse cross section of the population. In the United Kingdom, the National Health Service (NHS) provides services to more than a million people every 36 h [14] and healthcare is provided to individuals from across the socio-demographic spectrum. Secondly, there is a moral (and economic) argument that rather than just treating the consequences of obesity, healthcare providers should engage with efforts to actively prevent weight gain by supporting healthy choices and limiting access to unhealthy choices in their own premises [15]. Thirdly, many workers based in healthcare organisations are overweight or obese [16–18], which is likely to have a negative impact on employee health, performance and turnover [19]. These workers may benefit from the provision of an environment that is more supportive of health. Fourth and finally, there is capacity for improvement as modern food retail environments in hospitals are only ‘minimally conducive’ to healthy eating [20] with many unhealthy foods offered for sale in hospital cafes, shops and vending machines [21].

Where interventions that change the availability of healthy and unhealthy foods have been tested within the hospital setting, many have reported beneficial results. A recent systematic review identified 18 studies which assessed the impact of structural changes to the healthcare food environment on the dietary behaviours of staff, concluding that changes in the availability of healthy and unhealthy foods are among the measures most likely to be effective [22]. At the national level, healthcare organisations have in recent years implemented reforms designed to reduce the availability of unhealthy foods. In England, a proportion of healthcare providers’ income is now contingent upon ensuring that (amongst other targets) at least 80% of the confectionary and sweets available in hospitals do not exceed 250 cal and that < 10% of the drinks sold (by volume) are sugar sweetened [23]. In Scotland, mandatory nationwide standards were introduced (Healthcare Retail Standards; HRS [24];) between 2015 and 2017 which force hospital based retailers to ensure that at least 50% of all foods and 70% of all drinks available meet enhanced nutritional standards.

A recently published evaluation of the effects of the Scottish Healthcare Retail Standards [25], reports that the standards have been associated with a significant reduction in the purchasing of unhealthy foods and drinks. However, the observed reduction in purchasing of unhealthy products was not associated with a comparable increase in purchasing of healthy products, indicating that consumers have not swapped unhealthy for healthy products and may instead be shopping elsewhere. This possibility is supported by the results of a recent systematic review which demonstrates that structural food choice interventions in the workplace (while changing dietary behaviours at work), have no effect on the body weight of staff: workers who purchased and ate less at work compensated for this by eating more at a later opportunity [26]. Similarly, a recent audit of food purchasing in NHS hospital sites across one large UK city demonstrated that even where healthy options were readily available, most of the top selling items were unhealthy product lines – i.e. that many consumers were sticking to the unhealthy products that remain available [21].

If consumers are hesitant to make healthy changes to their purchasing behaviour even in the context of large increases in the relative availability of healthy options, further intervention may be required. One way to achieve this may be to introduce point of purchase prompts designed to prompt consumers to consider healthier options at the moment of choice. Information based cues, i.e. written or pictorial cues that convey information about a product at the moment of choice, have the potential to change consumers’ food choices [27] and interventions which have supplemented changes in availability with prompting information suggest that prompts may be sufficient to move purchasing away from highly unhealthy products [28].

Cognitive informational prompts or nudges in the context of healthy eating can be broadly divided into those which provide factual information with the intent of informing the consumer (e.g. calorie, fat or sugar content labelling) and those which provide some form of visual heuristic or shortcut that enables consumers to make an evaluative judgement about the relative healthiness of a product (e.g. traffic light labelling, symbols which denote healthy choices etc). While a recent large meta-analysis of 96 field studies concludes that cognitive nudges overall have smaller beneficial effects than nudges which are affectively or behaviourally targeted [29], several recent studies in the hospital setting have reported beneficial effects of evaluative traffic light labelling on the purchasing of healthier foods [30, 31].

One key feature of traffic light labelling which may explain this relative success is that such labels provide easy to digest, summary information. Most consumers only look at a small subset of the options available to them [32], and so nudges that visually highlight the better options ‘at a glance’ are likely to be useful. However, traffic light labelling is limited by the fact that the labels are applied to individual products and must still be compared from one product to the next, and that labels do not offer useful guidance in situations where all items within a particular category (e.g. chocolate bars) are likely to be labelled the same way (red).

The present study tests the effects of point of purchase prompts (PPPs) specifically designed to facilitate product comparison by displaying products in order from highest to lowest energy content. This type of prompt combines traditional nutrition information with a structure that enables consumers to determine at a glance the relative healthiness of any product in comparison to all other items on offer. To determine their effects on choice, these PPPs were introduced to hospital food shops to investigate whether they were effective in reducing the average calorie / fat / sugar content of snacks purchased by staff, patients and visitors. As future implementation would depend in part on ensuring continued profitability in host sites, the study also investigated whether the PPPs produced any change in average consumer spend or in the total number of products sold.

## Methods

### Design

A 2-arm cluster randomised controlled trial comparing the nutritional content of snacks purchased from hospital shops randomised (1:1) to display PPPs (intervention) or not (control). PPPs were installed and purchasing data were requested from the operating retailer for all sites over a 6-month period: the 12 weeks prior to installation (baseline) and the 12 weeks following installation (follow up). The trial protocol is available on request from the study authors.

### Setting

NHS Scotland currently has around 300 acute and community hospitals serving >5million people [33], many of which include some on-site food retail provision (e.g. shops, cafes, vending machines). Within these hospitals are 70 food retail shops [25], and the present study was set in those* (*n* = 30) owned by one national retailer (the Royal Voluntary Service; *full shops with regular opening hours, not including smaller kiosks with restricted hours). Shops were eligible for inclusion if they; sold snack food; were located in a hospital; and were accessible to staff, patients and visitors. Sample size was pragmatically determined based on number of sites operated by our collaborating retailer partner which met the study inclusion criteria. Included shops were located in urban and community hospitals in 9 different health boards; NHS Highland (*n* = 1), NHS Grampian (*n* = 5), NHS Tayside (*n* = 1), NHS Forth Valley (*n* = 4), NHS Fife (*n* = 2), NHS Borders (*n* = 1), NHS Lothian (*n* = 9), NHS Lanarkshire (*n* = 2), NHS Greater Glasgow & Clyde (*n* = 5). Sites were randomly allocated to intervention or control and individually tailored PPP interventions (shelf signs) were prepared for sites allocated to intervention (depicting the available product range in each location).

### Target behaviour

The intervention aimed to reduce purchasing of unhealthy single serve snacks, that is items which are typically purchased and consumed in their entirety and which do not typically constitute a meal. These foods were targeted in the current intervention because (a) snacks are typically supplementary to the core diet, (b) snack foods were available in all included hospital food retail sites, (c) single serve products are typically consumed in their entirety (unlike e.g. family packets of biscuits) so it is easier and more appropriate to infer amount consumed following purchase, and (d) there is enough nutritional variance within this category (e.g. from fresh fruit to pre-packed cakes and traybakes) for a successful behaviour change intervention to make a health relevant difference in consumption. The products which fell into the single serve snack category included confectionary, fruit, dried fruit snacks, crisps, savoury snacks, cereal bars, and pre-portioned cakes, muffins and traybakes (*n* = 230 different product lines in total). Three types of single serve products were removed from the data set prior to analysis; chewing gum (as it is not consumed); products designed for customers with special dietary needs such as diabetic sweet ranges (as customers purchasing these items often cannot change to an alternative); and seasonal items such as Easter eggs which were only temporarily available during the measurement period.

### Intervention

**Fig. 1:**
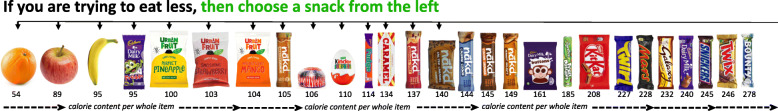
Sample point of purchase prompt (PPP)

The intervention was a Point of Purchase Prompt (PPP) - a sign to be displayed at eye level on shop shelves - designed to prompt purchasers to choose a healthier snack at the moment of choice. The PPP used in the present intervention was a sign displaying all of the available single-serve snacks in order from lowest calorie on the left to highest calorie on the right (see Fig. 1 for an example). It was developed by a multi-disciplinary team of psychologists, public health scientists, and nutritionists (Scottish Government Chief Scientist Office project CZF/1/37), and has been shown to significantly reduce the proportion of high calorie snacks purchased in a single hospital café in a previous pilot study [34]. The signs reduce the need for effortful cognitive processing in four ways. First, they prompt consumers to consider dietary intentions at the moment of choice, reducing the need for prospective memory about current goals. Second, the wording follows the format of an implementation intention (an ‘if-then’ plan [35];) reducing the need for advance planning of appropriate actions in light of current goals**.** Third, the layout and use of arrows capitalises on peoples’ tendency to show attentional biases to stimuli presented in left visual field [36] and towards items marked as goal-relevant [37], directing selective attention towards healthy items and facilitating inhibition of unhealthy items. Fourth, a single whole value (calorie content) is displayed for each product, and products are ordered according to these values to allow easy comparison between items, reducing the need for information to be held in working memory during choice. The intervention has been coded as containing 8 distinct behaviour change techniques [38]: instruction on how to perform a behaviour (BCT 4.1), salience of consequences (BCT 5.2), action planning (BCT 1.4), prompts/cues (BCT 7.1), behaviour substitution (BCT 8.2), conserving mental resources (BCT 11.3), distraction (BCT 12.4), and adding objects to the environment (BCT 12.5). Unique PPPs were produced for each of the study sites following the same template but with displayed items tailored to reflect the available product range in each location.

### Randomisation and blinding

Sites were classified according to their annual revenue (3 levels; low, medium, high) and randomised (1:1) to intervention (*n* = 15) or control (*n* = 15) by JA using block randomisation following the procedure outlined in [39] to minimise imbalance between the two groups in terms of revenue (as a proxy for unit size). All sites randomised received the intended treatment and were analysed for the primary outcome. Shop staff were not blinded to group assignment but data providers were. Data were analysed using mixed effects multi-level regression models.

### Outcome variables

The primary outcome was the average energy content of products purchased each day (kcals per product per day) during the trial. Secondary outcomes were average fat and sugar content of products purchased each day, average cost of each product purchased, and number of products purchased per day.

### Data collection

No data was collected directly from purchasers (who were a combination of hospital staff, patients and visitors). Rather purchasing data was collected directly from the retailer at the day level, i.e. number of each product sold per day.

*Nutritional information* about each product was collected from product packs during visits to sites and / or direct from suppliers. Where this was not possible (e.g. for fruit), information was taken from standard published nutritional information. Data on the energy content (kcals), fat content (g) and sugar content (g) per 100 g was extracted along with pack size and used to calculate the total energy/fat/sugar content of each whole single serve product in its entirety.

*Purchasing data* were obtained directly from the retailer which owned and operated the 30 participating sites. Data on every item purchased each day during the study period (January – July 2019) were provided (including date of each purchase, location of each purchase, price, brand and size of each purchase) and information pertaining to target items (single serve snacks) was extracted and used in the subsequent analyses.

### Procedure

Each participating site was visited by a researcher and the details of the available product range and site layout were recorded. This information was then used to create individually tailored intervention PPPs for each location. The PPPs showed only the product range available in each location and all used an identical template. PPPs were designed to fit in with existing shop signage by fitting onto shelf price rails. To reduce the likelihood that they were removed or damaged during restocking, the PPPs were installed on hinged brackets which enabled them to fold back completely when required to access the backs of shelves. All PPPs were printed at high resolution and covered in durable plastic to ensure that they were wipe clean and conformed to hospital infection control guidelines. Managers and staff in each location were informed by their regional manager that their organisation was participating in a research study about food choice and that some sites would have additional signage installed for a period of 12 weeks. Participating food retail sites were randomly allocated to intervention (PPPs) or control (no PPPs) in early 2019 and a researcher visited all those allocated to intervention in March 2019 to install PPPs at eye level on the shelves which contained the single serve snacks. As sweet and savoury snacks were sold in different parts of stores, two PPPs were installed in each intervention site – one on the sweet snack shelves and one on the savoury snack shelves. Sites randomised to control received no additional signage. The retail organisation provided the research team with details of every item purchased between January and July 2019 in the 30 participating retail units (both intervention and control). Purchasing data from the 12 weeks prior to the installation of interventions was used as baseline data and data from the 12 weeks after installation as follow up data. The trial ended in August 2019 as agreed with the host retailers. During the measurement period, a member of the research team contacted each intervention site to check the signs were still present and unobscured. While one site reported a sign coming loose, it was re-attached the same day so the intervention was delivered as planned across all sites.

### Analysis

Initially, a partial correlation matrix was computed to examine the relationship between various nutritional aspects of the product range sold in shops. This used the calorie (kcal), sugar, fat, and price data for all available products, and partialled out product size (grams). To test our hypotheses, multilevel models were used that nested daily mean nutritional content per purchase (Level 1) within weeks (Level 2) within retail units (Level 3). Control variables entered prior to our predictor of interest were: weekday-weekend (0, 1) and, in order to control for the outcomes (daily means) being less precise on days with fewer purchases, the grand-mean centred number of purchases. In order to test differences in pre-post purchase data across the intervention and control arms, models included a binary fixed effect denoting the first 12 weeks or second 12 weeks (pre = 0; post = 1), a binary group fixed effect (intervention = 0; control = 1), and their interaction. Here, a difference in the pre-post fixed effect would indicate a change within the intervention group (i.e. where group = 0) and the interaction effect would indicate whether this change was different to any seen in the control group. The group effect would indicate the difference during the first period of observation (i.e. where pre-post = 0). All models included random intercepts, and used an autoregressive (AR(1)) structure to account for autocorrelation between observation days. Identical model structures were used to model daily (i) calories per purchase (Model 1); (ii) fat per purchase (Model 2); (iii) sugar per purchase (Model 3); and (iv) cost per purchase. The number of sales per day was treated as a count variable and analysed using a Generalised Estimating Equation (GEE) model assuming the negative binomial distribution. The GEE model contained the same control variables as indicated above for the multilevel model. All analysis was carried out using SPSS v25, with statistical significance determined using α = .05.

## Results

Overall, the dataset contained information on the purchasing of 1,107,087 items in 30 hospital shops (15 intervention, 15 control) over a 6-month period. All shops randomised provided data and were included in the analyses. These data are summarised in Table 1 below.

**Table 1:** Descriptive data of purchased products (price and nutritional content)

	Overall	Intervention		Control	
		Pre	Post	Pre	Post
	M (SD)	M (SD)	M (SD)	M (SD)	M (SD)
KCal	189.53 (14.48)	191.27 (16.38)	189.57 (15.78)	189.40 (13.43)	187.57 (11.00)
Sugar	17.05 (2.25)	17.25 (2.16)	17.12 (2.16)	16.92 (2.41)	16.86 (2.23)
Fat	8.27 (1.10)	8.31 (1.27)	8.24 (1.20)	8.32 (1.03)	8.20 (0.81)
Products Sold	251.68 (230.11)	212.78 (167.11)	224.97 (187.81)	273.15 (265.21)	303.71 (277.25)
Pence per purchase	78.20 (5.65)	78.80 (6.13)	77.95 (5.66)	78.34 (5.76)	77.62 (4.80)

As shown in Table 2, the calorie content of individual items was positively correlated with both sugar and fat content. Price per item in contrast was positively correlated with calorie and fat content but negatively associated with sugar content (i.e. more expensive items tended to contain more calories and more fat but less sugar).

**Table 2:** Partial correlation matrix showing associations between nutritional content for the available product range

	M (SD)	Range	Price^b^	KCal^c^	Sugar^d^	Fat^d^
Unit size^a^	46.58 (22.82)	15–133				
Price^b^	0.99 (0.46)	0.25–2.69	–			
KCal^c^	183.28 (83.53)	23–506	.304***	–		
Sugar^d^	13.55 (11.47)	0–51.4	−.227***	.340***	–	
Fat^d^	7.98 (5.58)	0–30	.249***	.881***	.101	–

Note. ^a^Unit size (g); ^b^ Price (£ Pounds Sterling); ^c^ Calorie content (KCal/unit); ^d^ Nutritional content (g/unit). * *p* < .05, ** *p* < .01, *** *p* < .001

### Nutrition outcomes

#### Average calorie content of purchased items

Purchases made from intervention sites were significantly lower in calories (by 1.84kcals on average) in the post-phase relative to the pre-phase (95% CI: − 0.83,-2.85, *p* < .001; Table 3). The interaction between group (intervention; control) and time (pre; post) indicates that the pre-post reduction in calories observed in the intervention group was significantly larger to that in the control group (*p* = .049).

**Table 3:** Multilevel model estimating intervention effects on daily mean KCal per purchase

					95% CI	
	γ	SE	t	p	Lower	Upper
Intercept	190.30	2.51	75.75	< .001	185.17	195.43
Group	−2.57	3.55	−0.72	.474	−9.83	4.68
Pre-Post	−1.84	0.52	−3.57	< .001	−2.85	−0.83
Group*Pre-Post	1.50	0.76	1.97	.049	0.01	2.99
Weekend	6.68	0.59	7.57	< .001	5.53	7.84
Purchases	0.02	0.00	6.62	< .001	0.01	0.02

*Note.* Group: 0 = intervention; 1 = control. Pre-post: 0 = pre; 1 = post. Weekend: 0 = midweek; 1 = weekend; Purchases = number of purchases on day, centred at the grand mean

#### Average fat content of purchased items

There was no significant change in the fat content of purchases made from intervention sites (*p* = .07; Table 4), nor was the interaction between group (intervention; control) and time (pre; post) significant (*p* = .09).

**Table 4:** Multilevel model estimating intervention effects on daily mean Fat within each purchase

					95% CI	
	γ	SE	t	p	Lower	Upper
Intercept	8.22	0.20	40.17	< .001	7.81	8.64
Group	− 0.03	0.29	− 0.12	.906	−0.63	0.56
Pre-Post	−0.07	0.04	−1.84	.066	−0.15	0.00
Group*Pre-Post	0.10	0.06	1.71	.088	−0.01	0.21
Weekend	0.56	0.05	12.42	< .001	0.47	0.65
Purchases	0.00	0.00	6.39	< .001	0.00	0.00

*Note.* Group: 0 = intervention; 1 = control. Pre-post: 0 = pre; 1 = post. Weekend: 0 = midweek; 1 = weekend; Purchases = number of purchases on day, centred at the grand mean

#### Average sugar content of purchased items

Purchases made from intervention sites were significantly lower in sugar (by 0.18 g) in the post-phase relative to the pre-phase (95% CI: − 0.02, − 0.34, *p* = .03; Table 5), but the interaction between group (intervention; control) and time (pre; post) was not significant (*p* = .48).

**Table 5:** Multilevel model estimating intervention effects on daily mean Sugar within each purchase

					95% CI	
	γ	SE	t	p	Lower	Upper
Intercept	17.18	0.29	59.76	< .001	16.60	17.77
Group	−0.57	0.41	−1.39	.173	−1.40	0.26
Pre-Post	−0.18	0.08	−2.18	.030	−0.34	−0.02
Group*Pre-Post	0.09	0.12	0.70	.482	−1.55	0.33
Weekend	0.84	0.10	8,73	< .001	0.65	1.02
Total Purchases	0.00	0.00	10.03	< .001	0.00	0.00

*Note.* Group: 0 = intervention; 1 = control. Pre-post: 0 = pre; 1 = post. Weekend: 0 = midweek; 1 = weekend; Total Purchases = number of purchases on day, centred at the grand mean

### Sales outcomes

#### Number of items purchased

There was no significant difference in the number of individual products purchased per day from intervention sites in the post-phase relative to the pre-phase (95% CI: 0.84–1.21, *p* = .90; Table 6), nor was the interaction between group (intervention; control) and time (pre; post) significant (*p* = .64).

**Table 6:** Generalised estimating equation modelling intervention effects on daily mean purchase counts

					95% CI Exp (γ)	
	γ	SE	p	Exp(γ)^a^	Lower	Upper
Intercept	5.38	0.06	< .001	216.82	192.46	244.25
Group	0.28	0.10	.005	1.32	1.09	1.61
Pre-Post	0.01	0.09	.901	1.01	0.84	1.21
Group*Pre-Post	0.07	0.14	.637	1.07	0.81	1.41
Weekend	−0.69	0.02	< .001	0.50	0.48	0.52

*Note.* Probability distribution: Negative Binomial; Scale Parameter = 0.65; Group: 0 = intervention; 1 = control. Pre-post: 0 = pre; 1 = post. Weekend: 0 = midweek; 1 = weekend; ^a^ Exp (γ) can be interpreted as the percentage increase (values > 1) or decrease (values < 1) in the purchase count for each 1-unit increase in the predictor

#### Average spend per item

Purchases made from intervention sites cost significantly less (by 0.9p on average) in the post-phase relative to the pre-phase (95% CI: − 0.46, − 1.32, *p* < .001; Table 7). There was also a significant interaction between group (intervention; control) and time (pre; post) indicating that this reduction was significantly different to that in the control group (*p* = .03).

**Table 7:** Multilevel model estimating intervention effects on daily mean price per purchase (pence)

					95% CI	
	γ	SE	t	p	Lower	Upper
Intercept	78.37	0.93	84.21	< .001	76.47	80.27
Group	−0.67	1.32	−0.51	.617	−3.35	2.02
Pre-Post	−0.89	0.22	−4.06	<.001	−1.32	−0.46
Group*Pre-Post	0.71	0.32	2.21	.027	0.08	1.35
Weekend	2.16	0.23	9.31	< .001	1.71	2.62
Total Purchases	0.00	0.00	2.06	.039	0.00	0.00

*Note.* Group: 0 = intervention; 1 = control. Pre-post: 0 = pre; 1 = post. Weekend: 0 = midweek; 1 = weekend; Total Purchases = number of purchases on day, centred at the grand mean

## Discussion

Point of purchase prompts (PPPs) that cognitively simplified snack choice were associated with a small but significant reduction in the average calorie and sugar but not fat content of snacks purchased from hospital shops. The intervention had no effect on the number of products sold. It did however slightly reduce the average amount spent per item, likely reflecting the fact that across all available products, higher calorie items were slightly more expensive than lower calorie items and the intervention reduced calories purchased.

While the observed effects at the purchase level are modest (~2kcals/0.2 g sugar reduction per item), an average reduction of this size scaled to the > 1 million snacks purchased from the 30 participating shops during the period of the study, equates to purchasing of approximately 2 million fewer calories and 220,000 fewer grams of sugar. For individual customers, this effect will be insufficient to have a direct impact on health. However, using such interventions at scale to take calories out of the purchasing and consumption cycle may in time accrue beneficial effects that are sufficient to offset the prevailing pattern of population level weight gain.

Small changes to dietary intake are likely to be a feasible and practical way of slowing or preventing ‘incremental weight gain’ at the population level [40–42]. Most people in the population gain weight gradually over time by consistently accruing a small positive energy balance. Analysis of longitudinal cohort data suggests that the positive energy balance associated with the average weight gain trajectory equates to just 10kcals of excess consumption per day [43]. Every intervention that reduces the calorie content of food choices, by even a small amount, is therefore potentially useful in preventing gradual weight gain as long as people do not compensate for this reduction by choosing and consuming more calorific foods at other times. Compared to larger, more dramatic changes in diet, small changes to purchasing and consumption are likely to be more realistic, acceptable and feasible for people to achieve. Evidence from weight loss studies suggests that ‘small change’ interventions can produce clinically significant weight loss (5% of body weight), and critically, that this weight loss is maintained over time [44–46].

The Lancet obesity series called for researchers to provide opportunities for consumers to re-assess existing unhealthy preferences at the moment of choice [47]. The present study does this by providing a prompt which cognitively simplifies and highlights healthy food decisions at the moment of choice. Many food purchases are made ‘in the moment’ [48] with little or no conscious awareness [5–7]. Even when nutritional information is technically available on packs, it is not necessarily salient or accessible to consumers at the moment of choice, particularly when they are faced with a large array of possible options. A high percentage of consumers, and particularly those with lower levels of education, mistake serving size information for pack size information and struggle to compare multiple products with different characteristics [49, 50]. Interventions like the present one, which reduce the cognitive demands of choice and make it easier to compare products, may help consumers to process and act on relevant information that they would otherwise disregard. While not directly tested in the current study, previous testing of the present intervention found that people with lower levels of cognitive resource (i.e. those who might be expected to experience more difficulties when planning and enacting goal-directed actions in daily life) were more likely than others to make healthier purchases in response to seeing the intervention [34], indicating that the intervention is of most use to those with the greatest capacity to benefit.

While the present study uses signs to present information about the nutritional content of available products to consumers, the PPPs differ from traditional labelling interventions in one key way. By ordering the available products from left to right according to their energy content, consumers can see at a glance how relatively ‘good’ or ‘bad’ different choices are when compared with all of the other possible options. Cognitively, this provides useful summary information that may not be available to consumers relying on traditional food labels where each individual labelled product must be viewed in isolation or effortfully compared to others of interest one at a time. Indeed, a recent systematic review of choice architecture interventions in healthcare settings concludes that food labelling (usually in the form of calorie or fat content labelling) is not sufficient on its own to change purchasing behaviour [22]. It is possible that labelling is necessary but not sufficient to change purchasing unless additional tools (like the present PPPs) provide consumers with an easy way to synthesise the information in a way that lets them readily compare the available options.

### Strengths and limitations

This study tested the effectiveness of a theory-based, successfully piloted and simple point of purchase intervention in multiple sites across nine different UK health boards. While great strides have been made in recent years to improve the availability of healthy foods in hospital shops, recent studies suggest that top selling products in hospital shops are still often unhealthy [21] and that consumers consistently shop ‘within category’, i.e. might move from high fat crisps to lower fat crisps, but are unlikely to switch from purchasing crisps to fruit [25]. Consequently, pragmatic interventions like the present PPP, which aim to help consumers make small changes to purchasing while retaining the flexibility to choose according to preference stand to play a valuable role.

The present study is not however without limitations. As in any real-world trial, the intervention had to be designed to work in the host context. In the present example, this meant that the PPPs had to be designed and manufactured to fit into the space available within the host retail units. The layout of these units dictated that signs could typically not be wall mounted, and floor stickers were precluded by hospital infection control and cleaning policies. Consequently, the only remaining option was to shelf mount the PPPs. As the clearance between shelves was limited, signs had to be made smaller (averaging 50-60 mm in height) than they were in the original pilot study (approximately 100 mm in height). This will almost certainly have had an effect on the visual salience and readability of the signage and will likely have reduced the number of people who noticed and were able to read the contained information. In addition, the intervention signs had to compete with existing promotional signage (e.g. highlighting meal deals etc) which meant that they were less visually distinct than they were in the pilot study. It is possible that the effect of the intervention would be amplified if the signs were larger, more visible and presented in isolation.

A further limitation is the sole use of anonymous purchasing data. While this provides an objective and unbiased measure of purchasing, it does not rule out the fact that foods can be purchased but not consumed (e.g. purchased for others) or that people may compensate for healthier purchases by eating more later. It also limits the analyses that can be conducted. We were unable for example to test whether customers from particular groups (staff, patients, visitors, etc) were more or less likely to respond to the intervention. We were also only able to acquire purchasing data at the item level (i.e. how many of each particular item were sold, in each location on each day of the study). This meant that we were not able to look at clustering in purchasing (e.g. buying particular combinations of items) or at temporal patterns of purchasing in fine detail (e.g. purchasing variations by time of day). Similarly, it is not possible to determine whether the observed reduction in calorie / fat / sugar content of purchases reflects a small change in the purchasing of many customers or a large change in the purchasing of a minority of customers.

A final limitation is the number of retail units involved in the study. Several hospital-based retailers were not willing to host the intervention in their shops and our collaborating partner operated a limited number of eligible sites in Scotland. This naturally limited the number of sites we could enrol. However, as all sites operated by our partner retailer were included in the study (covering 9/11 mainland health boards in Scotland), the results are likely to be generalizable to other UK hospital based food retailers.

### Future directions

Future studies should aim to address the above limitations and to test the possible beneficial effects of physically re-ordering the available products on the shelves to correspond to their order in the PPP signage. Interventions that physically alter the position or proximity of target products in the environment have been recognised as choice architecture interventions likely to change behaviour [11]. In a recent study of snack choices from vending machines, Rosi and colleagues [28] arranged the available snacks into 4 sections from left to right (“healthy +”, “healthy −”, “unhealthy −”, and “unhealthy +”), finding that this manipulation reduced sales of “unhealthy +” snacks. Strategies which can reduce purchasing and consumption of the products at the more extreme end of the distribution in this way are likely to be well worth pursuing, given that a significant minority of snack options are extremely unhealthy ([21]; for example finding single-serve snack options with >600kcals, > 20 g fat, > 70 g sugar for sale in hospitals). Even if purchasing is simply driven away from the extreme endpoint of the distribution of available snacks, the effects are likely to be beneficial. Over time, customer rejection of and reduced demand for highly unhealthy products would be expected to shape the options that retailers provide, creating a gradual shift towards an even healthier range.

Similarly, future studies should investigate who changes their behaviour in response to prompts and who doesn’t, and in those who do, whether they make small within-category changes (e.g. choosing a chocolate bar with 200 kcals instead of one with 220kcals) or swap one type of snack for another (e.g. swapping chocolate for a cereal bar). Purchasing data alone suggest that customers in hospitals have responded in the former way in response to changes in item availability, but it is possible that PPPs may be able to prompt shifts from one category to another. One modelling study has suggested that simply replacing one unhealthy snack (e.g. chocolate bar) with one healthy snack (e.g. piece of fruit) per day would be sufficient to prevent approximately 6000 cardiovascular deaths per year in the UK [51].

## Conclusions

Simple, low cost, theory-based point of purchase prompts can produce small but significant reductions in the energy content of snack purchases made from hospital shops. Small positive changes to food purchasing and consumption may play a valuable role in controlling population level weight gain and so future research should focus on determining how, when and why such interventions work so that their effects can be optimised.

## Data Availability

The datasets generated and/or analysed during the current study are not publicly available due to their commercial nature but are available from the corresponding author on reasonable request and with permission of the Royal Voluntary Service.
